# In Memoriam

**Published:** 1874-02

**Authors:** Alex. Erskine, B. W. Avent, W. V. Taylor, E. Miles Willett

**Affiliations:** Com.; Com.; Com.; Secretary


### IN MEMORIAM.

With deep regret we are called upon to chronicle the death of a long experienced and an able *confrere* in medical journalism, Dr. Samuel Worcester Butler, senior editor and proprietor of the *Medical and Surgical Reporter*.

Dr. Butler was born at Brainard, East Tennessee, May 1, 1823, and died, at his residence in Philadelphia, on the 6th of January, 1874, after a lingering illness, of phthisis pulmonalis. He studied medicine in the office of his father, and graduated at the University of Pennsylvania, in the year 1850. He settled in Burlington, New Jersey, and was soon invited by Dr. Joseph Parrish, the editor, to an assistant editorship of the *New Jersey Medical Reporter*, of which, in 1854, he became sole editor and proprietor. In 1858 he removed his residence to the city of Philadelphia, where he continued his journal as the *Weekly Medical and Surgical Reporter*, with ever increasing success.

---

We have receiced a "Memoir of the Life and Character of Dr. Charles F. Fahs; read before the Selma Medical Society, on the 1st Monday in January, 1874, by James Kent, M.D."

Dr. Fahs was born near York, Penn., 10th of October, 1827, and died near Griffin, Ga., 17th November, 1873, æt. 46. He studied medicine with Dr. Kerr, of York. In 1848 he became the private pupil of Dr. George B. Wood, of Philadelphia. He graduated in April 1850, at the University of Pennsylvania, and in 1851 passed his examination, at the head of his class, for commission of Assistant Surgeon in the United States Navy. "In December 1861, he reached Boston Harbor, and at once forwarded his resignation as full Surgeon, to which rank he had been promoted while on the coast of Africa. The United States Government, deeming his conduct disloyal, would not allow him to resign, and finding all efforts in this direction unavailing, he determined, without delay, to share the fortunes of his adopted State, Virginia. After many difficulties, and narrow escapes, and much adventure, he succeeded in making his way through the Federal lines, near Washington city, crossed the Long Bridge, and gained the Confederacy, reporting at Richmond to Secretary Mallory, the Secretary of the Navy, who at once presented him a commission as full Surgeon in the Confederate Navy, and ordered him to report for duty at New Orleans. After the surrender of New Orleans he was appointed to the Navy Examining Board, at Richmond. When the Naval foundry in this city was completed and put in operation, he was ordered on duty here and remained at this post until the war closed; since which time he has practiced his profession in this city."

Dr. Kent pays a graceful tribute to the attainments and character of his departed professional brother.

---

OUR DECEASED PROFESSIONAL BRETHREN OF MEMPHIS.—At a meeting of the members of the medical profession, held in the parlors of the Peabody Hotel, in Memphis Tennessee, on the 7th of November, 1873, Dr. I. H. Pittman was called to the chair, and Dr. E. Miles Willett appointed secretary.

Drs. A. Erskine, B. W. Avent and W. V. Taylor, having been selected as a committee for the purpose of presenting resolutions expressive of the sentiments of the assemblage, reported the following, which were unanimously adopted:

WHEREAS, It has pleased Almighty God, the great Ruler and and Arbiter of the destinies of men, to visit our city with a malignant pestilence, which has swept away, in its desolating march, seven of our professional brethren, and friends, viz: Drs. Crone, Minor, Kennon, Hatch, Blount, Freeman and Williams; therefore, be it

*Resolved.* That we, who have been so fortunate as to have passed safely through its ravages, do deplore and mourn their fall with a true and manly sorrow.

*Resolved,* That, in their death, the medical profession of Memphis, and the profession throughout the land, have sustained losses immeasurably great.

*Resolved,* That we feel a common sympathy and a personal affliction, the result of a common sorrow, and the rupture of the tenderest ties of sympathy and love.

*Resolved,* That humanity, affected and bereaved, weeps over their fall. On its altar they, with manly devotion, laid down their lives.

*Resolved,* That the city of Memphis could have sustained no greater sacrifice than the death of her noble and gifted physicians.

*Resolved,* That we tender to the families of the deceased our sincerest sympathies in their irreparable loss.

ALEX. ERSKINE, M.D.,<br>

B. W. AVENT, M.D., } Com.<br>

W. V. TAYLOR, M.D.,

E. MILES WILLETT, M.D., Secretary.

